# Autoimmune and autoinflammatory mechanisms in uveitis

**DOI:** 10.1007/s00281-014-0433-9

**Published:** 2014-05-24

**Authors:** Richard W. Lee, Lindsay B. Nicholson, H. Nida Sen, Chi-Chao Chan, Lai Wei, Robert B. Nussenblatt, Andrew D. Dick

**Affiliations:** 1 National Institute for Health Research Biomedical Research Centre at Moorfields Eye Hospital NHS Foundation Trust and UCL Institute of Ophthalmology, University Hospitals Bristol NHS, Foundation Trust, and University of Bristol, Bristol, UK; 2 School of Clinical Sciences, University of Bristol, Bristol Eye Hospital, Low Maudlin Street, Bristol, BS1 2LX UK; 3 Laboratory of Immunology, National Eye Institute, National Institutes of Health, Bethesda, MD USA; 4 State Key Laboratory of Ophthalmology, Zhongshan Ophthalmic Center, Sun Yat-sen University, Guangzhou, China

**Keywords:** Uveitis, Autoimmunity, Autoinflammation, Macrophages, T lymphocytes, Immunotherapy

## Abstract

The eye, as currently viewed, is neither immunologically ignorant nor sequestered from the systemic environment. The eye utilises distinct immunoregulatory mechanisms to preserve tissue and cellular function in the face of immune-mediated insult; clinically, inflammation following such an insult is termed uveitis. The intra-ocular inflammation in uveitis may be clinically obvious as a result of infection (e.g. toxoplasma, herpes), but in the main infection, if any, remains covert. We now recognise that healthy tissues including the retina have regulatory mechanisms imparted by control of myeloid cells through receptors (e.g. CD200R) and soluble inhibitory factors (e.g. alpha-MSH), regulation of the blood retinal barrier, and active immune surveillance. Once homoeostasis has been disrupted and inflammation ensues, the mechanisms to regulate inflammation, including T cell apoptosis, generation of T_reg_ cells, and myeloid cell suppression in situ, are less successful. Why inflammation becomes persistent remains unknown, but extrapolating from animal models, possibilities include differential trafficking of T cells from the retina, residency of CD8^+^ T cells, and alterations of myeloid cell phenotype and function. Translating lessons learned from animal models to humans has been helped by system biology approaches and informatics, which suggest that diseased animals and people share similar changes in T cell phenotypes and monocyte function to date. Together the data infer a possible cryptic infectious drive in uveitis that unlocks and drives persistent autoimmune responses, or promotes further innate immune responses. Thus there may be many mechanisms in common with those observed in autoinflammatory disorders.

## Overview of uveitis: clinical and standard concepts

Survival depends on the pivotal sense of vision. Many pathologies affect vision and the eye, and almost all involve the immune response at some level. The function of the immune system in the eye is critical; correspondingly, there are active mechanisms in place to preserve immune homeostasis. When these are disrupted, frank inflammation ensues, which is clinically manifest as uveitis.

Uveitis is defined as inflammation of the vascular uveal tract of the eye, including the iris, ciliary body, and choroid; however, adjacent structures such as the retina, optic nerve, vitreous, and sclera may also be affected. Therefore, in practice any intraocular inflammation involving compromise of the blood ocular barrier is considered to be in the same group of disorders. Clinically, uveitis is classified anatomically as anterior, intermediate, posterior, or panuveitis, depending on which anatomical structures of the eye are involved [1]. All these forms are characterised by an inflammatory cellular infiltrate, which ophthalmologists visualize directly in an office setting using a biomicroscope. The anterior chamber of the eye is filled with optically clear aqueous fluid, allowing the practitioner to clearly see infiltrating leukocytes that are counted and scored in accordance with standardized grading systems [1]. This also applies to vitreous gel, which occupies the posterior segment of the eye.

**Fig. 1:**
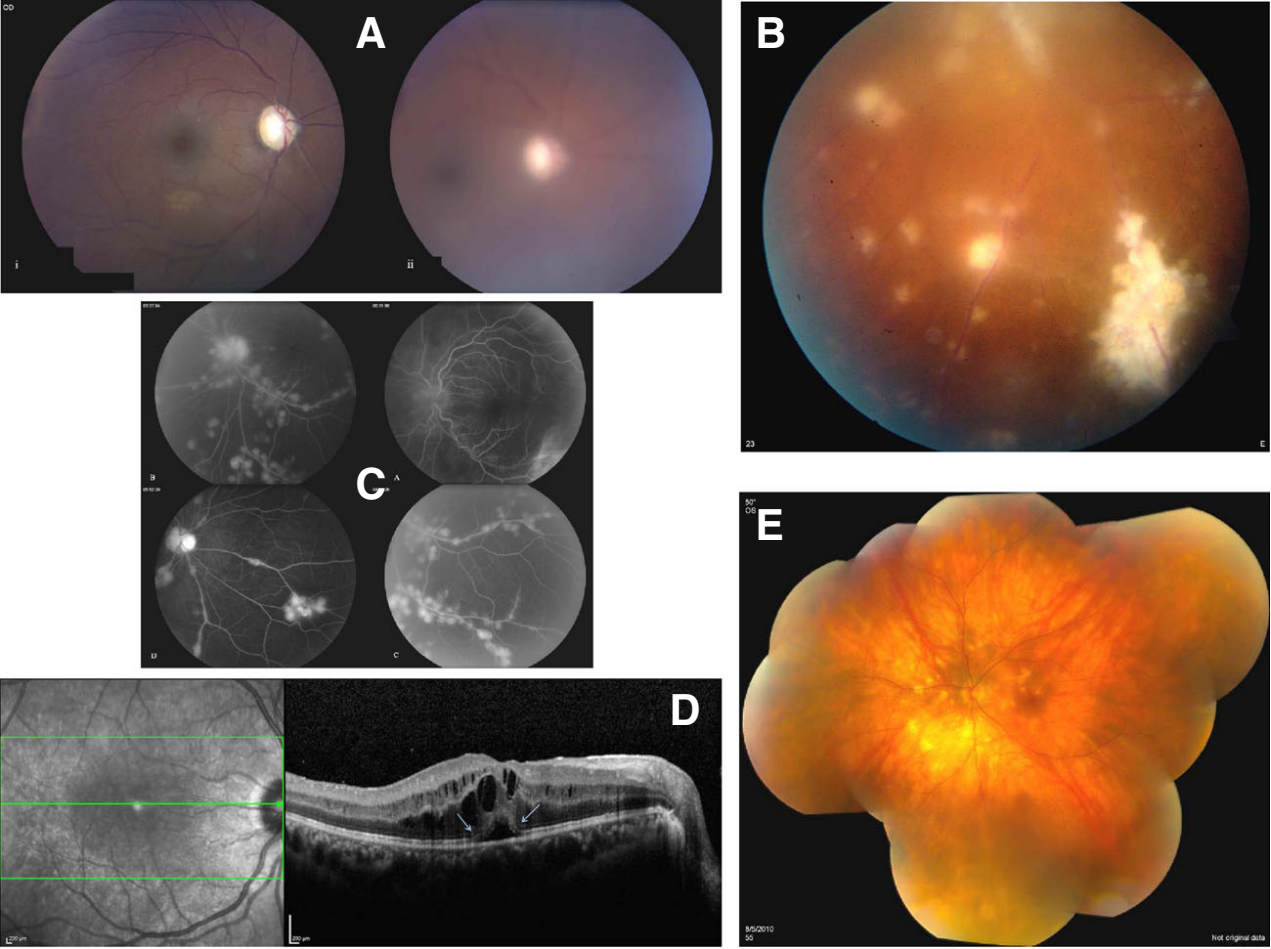
Diagnostic imaging depicting manifestations of uveitis. **a** Vitreous haze seen in the right eye of a 39-year-old African American female with sarcoidosis associated panuveitis (*i*, *left panel*) clears following treatment (*ii*, *right panel*). Please note that the borders of optic nerve and details of retinal vasculature are not clearly visible due to vitreous haze. **b** Peripheral fundus photographs of an African American male with neurosarcoidosis and panuveitis show significant perivascular exudates and chorioretinal granulomas. **c** Fluorescein angiogram of the same patient in **b** shows no staining in the very early phase but diffuse involvement of the entire retinal vasculature with staining of the exudates in early-mid phase (*upper right and lower left panels*) and leakage in late phase (*lower right panel*) is evident. **d** Spectral domain optical coherence tomography (SD OCT) of a 28-year-old Hispanic male with noninfectious uveitis and cystoid macular edema shows intra retinal cysts as well as subretinal fluid (*arrows*). Please note the detailed visibility of different retinal layers and the disruption of outer segment (*ellipsoid*) layer in the area of subretinal fluid (*arrow*). **e** Fundus photograph of the left eye of a 58-year-old Caucasian female with Birdshot chorioretinopathy shows multiple, deep, yellowish choroidal lesions scattered in the posterior pole, particularly nasal to the optic nerve

Protein exudates can result in an opacification of the usually clear ocular media, which is graded as flare in the aqueous or haze in the vitreous (Fig. 1a). Retinal and choroidal abnormalities are often localized, with clear foci of vascular inflammation or tissue infiltration (Fig. 1b). This clinical assessment is routinely augmented by ancillary tests such as fluorescein and indocyanine green angiography (Fig. 1c), which help determine the level of inflammatory activity in the retinal and choroidal tissues and consequent need for therapy. Recent advances in imaging technologies are now also generating high-resolution assessments in vivo of the retina in uveitic patients that approach histological clarity (Fig. 1d).

In 2010, WHO estimated that 285 million people were visually impaired; of these, 39 million were blind, and approximately 10 % was due to uveitis [2]. In the USA and Europe, uveitis accounts for 10–20 % of severe visual handicaps, and up to 10 % of blindness, in working age adults [3–7]. Uveitis may be caused by infections and/or autoimmunity. The relative proportion of causation is highlighted by geography; uveitis related to autoimmune disease is more common in developed countries, whilst overt infectious disease causes are more frequent in the developing world. Approximately 70–90 % of sight-threatening uveitis in developed countries is reported to be non-infectious [4, 8].

Non-infectious uveitis comprises a heterogeneous group of disorders diagnosed based on their clinical characteristics, which may be either confined to the eye or present together with systemic symptoms. Salient examples include birdshot chorioretinopathy (BCR), characterised by multiple small inflammatory lesions distributed throughout the retina and choroid (Fig. 1e); BCR’s association with the retinal protein S-antigen is well established [3, 9]. Although recent reports have demonstrated a systemic immune deviation in BCR [10, 11], clinically BCR is an isolated ocular pathology. In contrast, sarcoidosis and Behcet’s-associated uveitis and ankylosing spondylitis have clear systemic manifestations.

The clinical phenotype of non-infectious intraocular inflammation is replicated in experimental animal models that are driven by immune responses to self-antigen [12] The animal models support a role for autoimmunity, albeit experimentally inflammation is often shaped by the presence of mycobacterial protein. However, unlike other classical systemic autoimmune disorders, there are no clearly defined serological markers to assist diagnosis (e.g. autoantibodies) within majority of uveitis entities, except high HLA association (HLA-A29 and Birdshot chorioretinopathy). Nor are there markers in clinical use, predictive of either severity or prognosis. Nonetheless, 25 to 30 % of uveitis is associated with systemic autoimmune or autoinflammatory disease [4, 5], as described above.

To recapitulate uveitis in animals, the most commonly used model is experimental autoimmune uveoretintis (EAU) [13]. Although originally described in Guinea pig, intraocular inflammation may be induced in both rat and mouse. Disease model susceptibility is dependent upon strain and in turn MHC haplotype (e.g. H-2k mice). In particular in rats, the Lewis strain delivers a very susceptible aggressive monophasic disease and the mouse (H-2) on both B10 or BL/6 backgrounds and display after an acute response a chronic persistent disease. Traditionally, EAU has been induced via active immunisation with retinal antigens emulsified in complete Freund’s adjuvant. More recently, mouse models of spontaneous disease have been achieved by transgenic expression of retinal proteins (RBP-3) or neoantigens (HEL) with or without TCR transgenic T cells, or with human HLA [14–16]. The clinical features and pathology of these animal models bear remarkable similarity between some human conditions and mouse [17].

## Systemic versus local immune responses

Traditionally, uveitis has been categorized as either infectious or non-infectious, and there are obvious examples of each. That said, it is likely that both may co-exist. The immune response to an infection leads to a number of different possible outcomes. Uncontrolled immunity and/or unrestrained infection can lead to death. The successful elimination of a pathogen commonly leads to immune memory, but between these poles, infections may persist. Such chronic infections may either be controlled locally [18, 19] or lead to exhaustion of the immune response [20, 21]. These different outcomes illustrate a plastic component to immunosurveillance, with the potential for immune responses to adapt in response to alterations in the environment. In the context of autoimmunity, where autoantigen cannot be completely eliminated, the immune response may resemble that seen in persistent infection.

These dynamic considerations are relevant within the affected tissue as well as the systemic circulation. In the eye, this is especially pertinent because of the immune privileged nature of the tissue. Flowing from the seminal work of Medawar [22], the limited ability of tissues such as the eye and brain to reject non-MHC-matched transplants defined immune privilege. Historically, immune privilege was often interpreted as a lack of immunosurveillance. This absolute view is no longer appropriate [23–25]; instead, we recognise that immune cells visit healthy sites of immune privilege [26, 27]. Autoimmune diseases such as multiple sclerosis within these tissues radically alter the local dynamics of immune cell trafficking within the affected tissue [28]. The resulting remodelling may put the eye at risk of non-specific immune activation, precipitating clinical relapse; in humans, uveitis relapse has been associated with intercurrent infection [29].

In retina following inflammation, the ensuing rebalancing of immunosurveillance encompasses many immune cell types, including the well-established CD4^+^ and CD11b^+^ effector populations as well as NK cells, CD8^+^ cells, and B cells (J. Boldison, unpublished data). The importance of innate lymphoid cells in the eye is not yet known. The recent discovery that a population of CD3^+^CD4^−^CD8^−^ cells is both necessary and sufficient to recapitulate a model of the pathology of spondyloarthropathy [30] highlights the potential for small subpopulations of cells to organise local tissue inflammation. One relevant issue then is the fate of the lymphoid cells that are recruited to the eye. It is well established that during an immune response to a self-limiting pathogen, the majority of effector cells are eliminated from the systemic pool as the infection resolves [31]. Data in EAE models indicate that large numbers of CD4^+^ T effector cells die by apoptosis within the inflamed tissue [32, 33]; however, T cells may also traffic out of affected tissues, including the eye. This possibility is supported by our data that shows that treatment that arrests trafficking leads to a very rapid fall in the cell content of the eye [34, 35]. This result is consistent with a model in which immune cells exit the eye, as well as die in situ. In addition, studies evaluating other tissues such as the brain, lung, and gut [36–40] established that some effector cell populations (in these cases, CD8^+^ cells) take up long-term residence in tissues following infection. We have found this to be the case in EAU (J. Boldison and L. Nicholson, unpublished data), where infiltrating CD8^+^ cells of different phenotypes also show different patterns of migration. The expression of CD69, well-known as a marker of T cell activation, has more recently been associated with long-lived CD8^+^ tissue resident cell populations, whilst CD69-negative cells may recirculate more readily (Ref. [21] and J. Boldsion and L. Nicholson, unpublished data). The full spectra of mechanisms that regulate this process remain poorly understood. Stem cell niches which have been defined in bone marrow may be a useful corollary here. Complex interactions may be critical to maintaining these environments, where many different cell types can regulate niches both directly and indirectly, and where hematopoietic stem cell traffic in and out of the niches is reported [41].

## The role of T cells in driving adaptive immunity in the eye

The eye is affected by both autoinflammatory and autoimmune disease processes. Advances defining the molecular pathology of autoinflammatory conditions have led to an appreciation of the wide range of diseases in which inflammation is driven by genetic mutations affecting elements of the innate immune system. These conditions embrace a spectrum that is initiated by aberrant inflammation, but then includes adaptive immune elements [42]. The archetype for this type of autoinflammatory process in the eye is Blau syndrome, caused by gain-of-function mutations in the NOD2 gene that lead to increased basal nuclear factor κB (NFκB) transcriptional activation [43]. Patients present with early-onset granulomatous inflammation, skin rash, and camptodactyly. The lesions in the retina have a distinct and characteristic appearance, whilst an immunohistochemical analysis of skin and other peripheral granulomas reveals an abundance of CD4^+^ lymphocytes and CD68^+^ monocyte-macrophage lineage cells; there are fewer CD8^+^ lymphocytes, but large amounts of IFN-γ, IL-17, and IL-6 [44]. This example demonstrates that innate activation can lead to the involvement of adaptive immune cells in this autoinflammatory process, and illustrates the complexity that accompanies uncontrolled chronic immune activation.

Idiopathic autoimmunity arises following the activation and expansion of retinal antigen-specific T lymphocytes. Experimentally, the triggering event can happen at sites distant to the affected organ, although whether this occurs in human disease is rarely known. The dominant paradigm is that of a CD4^+^ T helper cell-driven process. The relevance of this to human disease is supported by the association of sympathetic ophthalmia and Vogt–Koyanagi–Harada disease with specific HLA class II alleles [45, 46], as well as the identification of ocular antigen-responsive T cells in both the peripheral blood and eyes of patients [47, 48]. The strong MHC association with autoimmunity arises both through the need for specific autoantigen presentation [49] and through the selection of a potentially pathogenic T cell repertoire [50].

Once potentially pathogenic T cells have been produced in the periphery, access to the immune privileged ocular environment may be under the control of vessel-associated antigen presentation, in a fashion analogous to that described in the brain [51]. But with their translation from blood vessels to tissue, local activation of antigen-responsive T cells provide the necessary signals that focus autoimmunity to the eye. Later, the same signals are also crucial for the activation of regulatory cells that limit the pathology due to inflammation within tissues.

When naive CD4^+^ T helper cells are activated, they reorganise transcriptional networks [52]. This leads them to assume different functional phenotypes, often characterised by the secretion of signature cytokines [53, 54]. Of the many genes that are regulated by this process, those that influence expression of chemokine receptors and subsequent patterns of tissue localisation are also important mediators of effector function. In rodent models of uveitis, immunisation with whole proteins or peptides induces a CD4^+^ T cell-dependent uveitis [55–57]. Studies using the transfer of CD4^+^ T cell lines and clones have confirmed that these cells are sufficient to initiate the autoimmune process in a number of different models [58–61]. By differentiating murine T cells in vitro and transferring them to naive hosts, the pathological potential of different T cell phenotypes has been evaluated [62]. In EAU, such studies identify both Th1 and Th17 T helper cells as important inducers of autoimmune disease [63]. When CD4^+^ T cells were purified from the retinas of animals with uveitis that was induced by peripheral immunisation, and then studied ex vivo, both Th1 and Th17 cells were found. The relative proportions of these populations change over time [28]. Cytokines produced by these cells condition the local microenvironment, and activate macrophages (especially IFN-γ produced by Th1 cells), recruit neutrophils, and potentially restructure the local environment (e.g. through IL-17 produced from Th17 cells; [64]). Differentiated T cell subpopulations also have a role in controlling local inflammation when they acquire a T regulatory phenotype. The normal ocular microenvironment favours differentiation to Foxp3^+^ regulatory T cells, but when the eye is already inflamed this is not the case [59].

Although CD4^+^ T cell-driven disease is the dominant paradigm in models of uveitis, studies have shown that it is possible to induce autoimmunity within the eye using antigen-specific CD8^+^ T cells [65]. Further, it has long been recognised that CD8^+^ T cell numbers increase during the course of experimental uveitis [57, 66]. As with Blau syndrome, a condition whose clinical ocular features manifest more with time, uveitis is the result of much more than the aberrant activation of a single cell type. For both CD4^+^ and CD8^+^ lymphocytes, their role within the tissue may change as disease progresses. In the EAU model, increasing evidence suggests there is persistent dysregulation of immunosurveillance of the retina following the induction of disease [67, 68]. These changes are a manifestation of the defining feature of the immune system: its ability to adapt to reduce the impact of subsequent infections after an initial encounter with a pathogen. From this perspective, it is informative to consider new data relating to the development of memory to viruses. Following infection, CD8^+^ memory populations take up long-term residence in tissues and adapt to those environments differently compared to residing in the lymphoid compartment. They then play an important role when tissues are re-challenged with the same infection [21, 37, 39].

The role of CD4^+^ and CD8^+^ lymphocytes in uveitis may therefore go well beyond the initiation of tissue destruction, and include the regulation of immunosurveillance of the local microenvironment by controlling the flow of cells in and out of the tissue. Regulating cell trafficking would limit local activation of recently recruited autopathogenic cells, whilst maintaining a local presence of CD8^+^ cells primed to respond to increases in antigen presentation.

## Control of myeloid cell function in the eye is influenced by tissue and cellular environment

The retina and choroid are furnished with a rich network of myeloid cells that create and establish immune tissue tone, and maintain immune health of these tissues [69]. This is especially pertinent when considering the function of the fragile neural retina, which evolved as a pivotal part of vision necessary for survival. The tight control of myeloid cell activation in the retina permits a continual survey of the environment, maintenance of scavenger function, and prevention of unwanted cellular and tissue damage. Whilst there are no data on the time course and extent of the inflammatory cell infiltrate during ocular disease in humans, results of studies in mice emphasize a predominance and persistence of macrophages throughout disease [28, 68]. The frequently cited ‘granulomatous’ uveitis (which heralds a clinical feature, not a pathological definition) illustrates the notion that there are large numbers of infiltrating inflammatory cells that aggregate and persist throughout course of disease, even after the acute phase of inflammation. This idea is further supported by studies of pathology in humans, and by interrogation of animal models that demonstrates discrete myeloid, macrophage, and T cell accumulations in later disease [17].

**Fig. 2:**
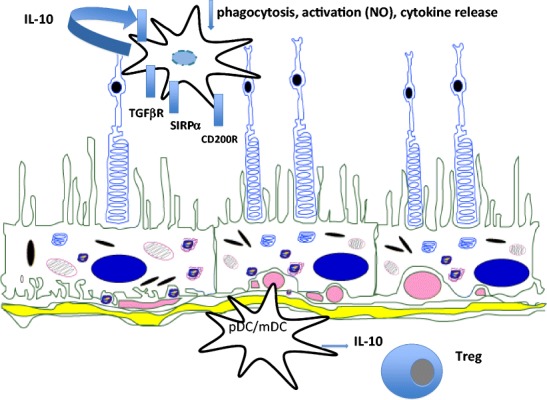
Regulation and setting of the threshold of myeloid cell responses within the retina and choroid. Microglia and choroidal myeloid cells (dendritic cells and macrophages) sense the environment and regulate inflammatory responses. The healthy tissue sets the threshold for response through inhibitory receptors (e.g. CD200R, SIRPα) or via the TGF-β rich environment. The regulation via neuronal cognate interaction is augmented by the regulatory functions of RPE, via mediators such as PD-1 and PD-L1 interactions, TGF-β secretion, and inhibitory peptides. The response to activation of myeloid cells is dominated by IL-10 release; whilst other pro-inflammatory cytokines are also produced, the default response is downregulation [71–75]

The dispute as to whether microglia contributes to onset of ocular inflammation [70] has to be balanced against their homeostatic role in maintaining a healthy retina, where the data is more compelling [71]. Microglia are networked throughout the retina, and display regulatory phenotypes and functions consistent with other tissue-resident macrophages elsewhere in the body [69] (Fig. 2). Thus, the tissue may set an activation threshold to prevent unwanted damage. For example, myeloid activation is controlled via cognate–receptor interactions, principally CD200R and its ligand, CD200; CD200 is expressed on neurons and endothelium [76–80]. Loss of receptor or ligand produced by genetically manipulating mice, or by blocking interactions in rats, results in an activated macrophage phenotype (NOS2-positive); following insult (either autoimmune or injury), a more aggressive disease phenotype results [72, 81]. Myeloid regulation can be reconstituted via ligation of receptor (e.g. by treatment with anti-CD200R monoclonal antibodies or by treatment with CD200Fc), which ablates and controls autoimmune retinal inflammation in EAU [73] and reduces consequences of injury [82]. This interpretation is supported further by similar observations in CNS [79]. Myeloid regulation operates in humans as well as mouse, and extends to control of mast cells and other tissue sites such as lung [83–87].

Overall, tissue damage in EAU is significantly attenuated when macrophages are removed [88, 89] or myeloid activation is blocked [90–92]. In EAU, compelling evidence indicates IFNγ-mediated macrophage activation that depends on TNF-α and functional TNFR1 results in high levels of nitric oxide, TNF-α, and IL-6. These mediators, in turn, generate lipid peroxidation and damage surrounding cells [93–96]. Experimentally, we consistently observe that the tissue is protected following neutralisation of TNF-α activity, or by reprogramming myeloid cell activation threshold with CD200R treatment. Not surprisingly, therefore, anti-TNF-α agents provide clinical benefit in human disease ([97–99], and see below).

Thus, the pivotal drive to tissue damage is via activation of the non-specific myeloid compartment. But even though classical IFN-γ-mediated macrophage activation is apparent, myeloid suppressor cell phenotypes that control T cell proliferation and targets have been observed. Such control is mediated through myeloid endoprostanoid receptors and nitric oxide [100, 101]. The critical balance of these responses serves to self-regulate via suppression of T cell function in situ and to clear danger; but tissue damage may result when this balance is not fully achieved.

A principal observation in murine EAU is the persistence of inflammation [102, 103], implying that the threshold of myeloid activation is not reset and homeostasis is not restored. In the presence of persistent T cell responses [28], the tissue remains vulnerable. A constant macrophage infiltrate remains, although in nearly all models the macrophages exhibit an alternative activation phenotype in later stages (as opposed to the earlier classical activation phenotype; [67]) that may be secondary to tissue remodelling. One result of a chronic immune cell infiltrate is persistent tissue remodelling contemporaneous with myeloid activation, of which one hall mark is angiogenesis. The angiogenic response during persistent tissue immune cell infiltrate requires an operative CCL2-CCR2 axis, but is also influenced by multifunctional matrix proteins, such as thrombospondin-1 (TSP-1) [67]. Subverting the angiogenic response (but without altering the initial inflammation and antigen-specific targeting of tissue) by knocking out matricellular proteins such as TSP-1 results as expected persistent disease (as observed in wild-type mice [104]) but notably results in increased angiogenesis (a detriment to retinal function as observed in neovascular diseases such as diabetic retinopathy and age-related macular degeneration). Moreover, macrophages secrete TSP-1 following TLR ligation; however, T cell activation regulates TSP-1: both Th1 and Th2 cytokines increase threshold for TLR-mediated TSP-1 production, and in their presence, less is secreted [100]. Together the results infer that there is matricellular control (e.g. TSP) of macrophage activation in terms of remodelling and angiogenesis during T cell mediated responses and whilst initial disease severity is not altered with loss of TSP, regulating tissue remodelling, (as determined by extent of angiogenesis) is perturbed.

Controlling the macrophage response certainly is a tractable therapeutic goal, as illustrated by the success of anti-TNF-α agents clinically. The ability of macrophages to respond to environmental, cytokine, and receptor signals provides adaptability in controlling inflammation and in restoring structure and function. Translation will remain challenging (given the plasticity of myeloid cells and how rapidly they adapt) when considering timing of treatment. In EAU there are other compounding influences to consider for therapy. For example, complement is activated during disease; whilst arguably not critical to development of inflammation [105, 106], suppressing or regulating complement diminishes EAU expression [107, 108]. The presumed mechanism of action is at the level of suppressing macrophage activation. Similarly, chemokine gradient support or perturbation can suppress or exacerbate EAU disease, where the myeloid compartments are being manipulated [109–113].

## Current understanding of pathology in humans, and future opportunities

Much of our understanding concerning the underlying pathology of ocular inflammatory disease has come from various animal models. Observations in humans have supported some, but not all, of the various mechanisms noted in animal studies, mostly pertaining to autoimmunity. However, globally infection remains a significant cause of uveitis. Infections can actively invade ocular tissue and result in an inflammatory process. These include toxoplasmosis, tuberculosis, syphilis, leprosy, and tularemia, as well as DNA viruses such as CMV, VZV, and HSV [114] with other possible pathogens emerging [115]. With some exceptions, the clinical evaluation of most patients in developed countries who are evaluated for an anterior uveitis will not demonstrate direct invasion of a pathogen into the ocular tissues. In most cases, aqueous samples taken from the front of the eye during active inflammation have shown inflammatory infiltrates, including T cells, B cells, and macrophages. Analyses of such samples also suggest active immune regulatory mechanisms, including FasLigand-induced T cell apoptosis and cortisol regulation of dendritic cell function [116–118]. However, an important issue is what initiates these inflammatory processes within the eye. Despite the absence of evidence of overt infection, experimental data suggest a central role for bacterial products. Anterior uveitis can be induced by injecting endotoxin (lipopolysaccharide) subcutaneously, intravenously, or intraperitoneally at a site far from the globe of the eye [119, 120]. A number of other bacterial products, including MDP (murinyl dipeptide), also have the capacity to induce an ocular inflammatory response [121]. These observations implicate innate immune activation and inflammasome activation via NOD [122]. In addition, 30 % of ankylosing spondylitis (AS) patients will have an anterior uveitis episode [123]. AS is associated with asymptomatic Crohn’s disease, a disorder in which bacterial products are strongly implicated [124]. It is quite possible that bacterial fragments act as adjuvants, activating the innate immune response and perhaps secondarily the adaptive immune system.

The types of immune cells in the eye and the cytokines they produce have been studied in intermediate and posterior uveitis. Proinflammatory cytokines such as IFN-γ, TNF-α, IL-1, IL-2, and IL-6 have all been reported in the eye during inflammation [125]. In other ocular conditions, such as Behcet’s disease, there may be an increase in cytokines such as TGF-β. Elevated IL-17 levels have been reported circulating in the blood of sarcoidosis patients [126, 127], and elevated levels of both IL-17 and IL-23 have been reported in BCR patients undergoing cataract extraction [10]. Immune responses have been further characterised in several other human disorders, either by evaluation of chorioretinal biopsies or by studying eyes removed for various reasons [128]. Chorioretinal biopsies, i.e. removing the choroid and retina together, are not performed routinely but have yielded much information, often helping in the choice of therapeutic options. Whilst T cells (including various subsets) predominate in the infiltrates, other inflammatory cells including B cells and macrophages have been identified. Studies of enucleated or post mortem eyes from uveitis patients have indicated upregulation of adhesion molecule expression on the retinal vessels, provided evidence of apoptosis of retinal cells after severe inflammation [129]. As noted above for EAU animal models, human tissue studies have also suggested a switch of macrophage subtypes in the retina of eyes from a classically activated to an alternatively activated phenotype [130].

In general, patients with uveitis exhibit a diversity of systemic immune responses. Microarray studies performed on the blood of uveitis patients demonstrated many different genotypic signatures, even amongst patients with the same clinical diagnosis [131]. To date, however, in vivo examination of the living eye, particularly the posterior pole, that would permit identification of immune cells is lacking.

One can only speculate as to the triggering mechanisms that lead to severe ocular inflammatory disease. Indeed, they may be multifactorial. This is clear from the immune profiling, which demonstrated at least four immune signatures [131]. Overt infection may not be the major force but it is reasonable to hypothesize a role for microbes acting as initiating or potentiating factors. In some cases, viral infections that have been cleared may have initiated immune responses that are then propagated by molecular mimicry (i.e. cross reactions with antigens found in the eye). Evidence of anamnestic responses to ocular antigens, particularly the retinal S-antigen, has been reported by many, in patients with both infectious and non-infectious processes in the eye [132]. A more subtle way in which microbes may be playing a role is their adjuvant effect, i.e. by shifting the balance from immune responses that are normally controlled by the immune system’s downregulatory mechanisms to ones that instead lead to overt disease. Two conceptual ideas may therefore be important in the development of uveitis. The first is the hypothesis that patients prone to developing an intraocular inflammatory response are those whose immune system has undergone a ‘loosening’ of the normal oversight of the immune system, as seen in immunosenescence. The second is that these changes may be very important in preventing or reversing the normally positive effects of parainflammation.

## Systems biology approach

It is apparent that uveitis is a complex disease involving multiple organs, often beyond the eye. The classical approaches to studying the pathogenesis of diseases, by focusing one or two candidate genes, have limited success in identifying disease-specific biomarkers, elucidating the complex molecular mechanism underlying uveitic disorders, and improving the clinical management of these sight-threatening diseases. The recent development of many high dimensional assays has allowed large-scale enumeration and quantification of millions to billions of endpoints [133]. Together with available computational/bioinformatic tools that manage, analyse, and integrate biological data, a comprehensive view of biological phenomena and disease process may be elucidated [134]. Despite extensive application of systems biology approaches in studying inflammatory diseases such as rheumatoid arthritis and multiple sclerosis, to date only a limited number of studies have investigated uveitis with high-throughput approaches.

Over the last decade, genome-wide association studies (GWAS) have been extensively used to identify disease-associated genetic variants in large patient cohorts [135]. The genetic risk of Behcet’s disease, amongst all uveitic disorders, has been well studied [136]. Using single-nucleotide polymorphism (SNP) arrays, Fei et al. [137] performed the first GWAS study of BD in 2009. In 2010, Remmers et al. [138] and Mizuki et al. [139] identified the MHC class I, IL23R-IL12Rβ2, and IL-10 loci as the major genetic risk factors in Behcet’s disease, using large patient and control cohorts from Turkey and Japan, respectively. Furthermore, more recent GWAS studies have elucidated many new genetic susceptibility loci, including CCR1, STAT4, KLRC4, CD40, HLA-B*51, and ERAP1 [102, 140–142]. Interestingly, many of the risk loci found in Behcet’s disease were shared with ankylosing spondylitis, psoriasis, and inflammatory bowel disease [143–147].

In addition to genetic susceptibility, the regulation of gene expression according to the environmental cues is also crucial in physiological and pathological conditions. Using DNA microarray technology, Usui et al. [148] revealed elevated expression of ICOS in Behcet’s disease. However, a genome-wide expression profiling study carried out by Li et al. suggested that rather diverse gene expression signatures exist amongst non-infectious uveitis patients [131].

Identification of globally dysregulated proteins in systemic and local inflammatory diseases became possible recently with a significant advance of technologies such as mass spectrometry, multi-parameter flow cytometry, and protein arrays [149]. Both ocular fluids and serum proteins from patients with Behcet’s disease [150, 151] and Vogt–Koyanagi–Harada disease [152] have been surveyed in multiple studies using mass spectrometry-based technologies. However, it is unclear how these proteins are related to intraocular inflammation and disease pathology. In addition, recent studies have demonstrated that data from single-cell-based analyses of peripheral blood cells using multiparameter flow cytometry can distinguish BD from sarcoidosis diseases, suggesting a broad application of high-dimensional proteomic datasets in uveitis diagnosis [153].

Obviously, systems biology approaches are rapidly generating large amounts of information on genomes, epigenomes, transcriptomes, and proteomes. However, appropriate bioinformatic tools for high-throughput data analysis are still needed [149]. Although novel biomarkers and targets that are amenable to drug development may potentially be identified by these global unbiased approaches, extensive validation is still warranted. With the utilization of game-changing technologies such as high-throughput sequencing technologies [154] and cytometry by time-of-flight [155] in clinical service, we expect that this multidisciplinary approach will accelerate biomarker discovery and drug development for uveitis.

## Targeting therapies for specific responses: perspectives and conclusions

Much knowledge has accrued through interrogating immunopathological processes in animal models of uveitis, with results that eloquently illuminate specific targets. As discussed, overall the models have demonstrated pivotal role for TNF-α, as well as activated CD4 T cells, their signature cytokines, and their ability to influence trafficking of cells. Approaches based on this body of knowledge are currently in early-phase, randomised controlled trials to treat uveitis. Alongside such developments, there are new therapeutic avenues to consider, some of which are common to many inflammatory diseases. First, autoimmune responses may be suppressed, i.e. by tolerance therapies [74, 156–159]. Second, specific T cell responses may be suppressed directly [160–162], or indirectly by suppressing antigen presentation or augmenting regulatory T cell responses [163, 164]. Third, non-specific tissue damaging responses may be disarmed by inhibiting macrophage function [72, 73, 82], by inhibiting cytokines [97–99, 165, 166], and by inhibiting trafficking of cells [34, 35, 167].

Whilst experimental results suggest reasons to hope, the limited clinical success to date is somewhat discouraging. Nonetheless, clinical outcomes raise important questions. How can outcomes be predicted? What are the major immunopathogenic drivers for each disease entity within spectrum of uveitis? How will patients respond to any given therapy? Close examination suggests very good evidence for success of anti-TNF-α agents in Behcet’s disease [166, 168–174] and other uveitides [99, 175]. Such results are encouraging in terms of translating findings in animal models to people. However, failures and gaps in our knowledge remain. For example, to date therapies have not been targeted to individuals. Trials are typically designed based on what are probably single types of uveitic disorders, but the subjects enrolled are likely to represent a heterogeneous group of disorders, all of which are grouped under the umbrella term uveitis. With the current lack of detailed understanding of separate clinical entities, it may be difficult to deliver and detect significant therapeutic responses. The experience with anti-IL-17 therapy is a case in point. Both animal models [176–180] and human observations [181, 182] clearly supported the logic of targeting this cytokine, particularly in Behcet’s disease. But the results of three trials using anti-IL-17 treatments [183] had no evidence for positive effects, and were disappointing. These results support increasing evidence inferring that the pathological mechanisms at play in Behcet’s disease are autoinflammatory, rather than autoimmune. That said, given that the studies did not attempt to match disease phenotypes with IL-17 biomarker expression, the trials did highlight the necessity of aligning treatments to the patient, and using biomarkers to target treatments to patients that are more likely to respond. Similarly, it is important to optimise the timing of treatment in disease evolution, as well as dose and route of administration of treatment. In the future, this may include combinatorial approaches. When knowledge of targets is optimal, success may become more evident, as we have appreciated from treating the spectrum of autoinflammatory conditions. For example, initial efforts to apply anti-TNF-α therapies in uveitis associated with Juvenile systemic granulomatous disease (Blau’s disease) [184–186] or anti-IL-1RA therapies in CINCA-NOMID [187] have been successful.

As discussed throughout, the challenge faced at the point of clinical presentation with many of the uveitides is the likelihood that they represent a spectrum of autoimmune conditions. These may be dominated by T cells or by antibodies, and/or reflect both autoimmune and autoinflammatory processes that drive innate responses. If largely driven by autoantibodies or B cells, or where vasculitis is predominant, then anti-CD20 therapy may be appropriate; indeed, in the right patients the therapeutic effect of anti-CD20 treatment with rituximab has been substantial [188–190].

Thus, there is a need to refine therapies for individual patients, and hopefully predict response prior to administration. Whilst there may well be common effector mechanisms in uveitis, such mechanisms remain influenced by epigenetic regulation or by inflammatory gene polymorphisms, skewing predicted drug effects [191–194]. Nonetheless, we can make progress. For example, in the current therapeutic paradigm of treating with corticosteroids prior to using steroid-sparing agents or specific biologic therapies, maximizing response rates requires us to address a common problem: up to 40 % of patients do not respond to corticosteroids. Corticosteroid resistance lies in part within the Th17 T cell population [195]. Such knowledge may facilitate our ability to target therapies designed to overcome the lack of steroid responsiveness at the time of presentation, and restore treatment responses in these patients [196–198].

Furthermore, advancing knowledge suggests opportunities to consider therapies that combine treatments and routes, i.e. by delivering one treatment to the eye alongside systemic delivery of another. For example, gene therapy might be used within the retina [199], whilst the systemic immune response is modulated simultaneously, such as via tolerance induction, by preventing cell trafficking, or by specifically neutralising cytokine or signalling pathways. Such strategies, which could be refined and targeted by taking advantage of appropriate biomarkers, could have the net effect of restoring immune health and homeostasis to the retina.
